# Detection of SARS-CoV-2 RNA in Bivalve Mollusks by Droplet Digital RT-PCR (dd RT-PCR)

**DOI:** 10.3390/ijerph19020943

**Published:** 2022-01-14

**Authors:** Andrea Mancusi, Federico Capuano, Santa Girardi, Orlandina Di Maro, Elisabetta Suffredini, Denise Di Concilio, Lucia Vassallo, Maria Concetta Cuomo, Maria Tafuro, Daniel Signorelli, Andrea Pierri, Antonio Pizzolante, Pellegrino Cerino, Giuseppina La Rosa, Yolande Thérèse Rose Proroga, Biancamaria Pierri

**Affiliations:** 1 Department of Food Security Coordination, Istituto Zooprofilattico Sperimentale del Mezzogiorno, Via Salute No. 2, 80055 Portici, Italy; andrea.mancusi@izsmportici.it (A.M.); federico.capuano@izsmportici.it (F.C.); santa.girardi@izsmportici.it (S.G.); orlandina.dimaro@izsmportici.it (O.D.M.); 2 Department of Food Safety, Nutrition and Veterinary Public Health, Istituto Superiore di Sanità, Viale Regina Elena 299, 00161 Rome, Italy; elisabetta.suffredini@iss.it; 3 Centro di Referenza Nazionale per l’Analisi e Studio di Correlazione tra Ambiente, Animale e Uomo, Istituto Zooprofilattico Sperimentale del Mezzogiorno, Via Salute No. 2, 80055 Portici, Italy; denise.diconcilio@izsmportici.it (D.D.C.); lucia.vassallo@izsmportici.it (L.V.); mariaconcetta.cuomo@izsmportici.it (M.C.C.); maria.tafuro@izsmportici.it (M.T.); daniel.signorelli@izsmportici.it (D.S.); andrea.pierri@izsmportici.it (A.P.); antonio.pizzolante@izsmportici.it (A.P.); strategia@izsmportici.it (P.C.); biancamaria.pierri@izsmportici.it (B.P.); 4 Department of Environment and Health, Istituto Superiore di Sanità, Viale Regina Elena 299, 00161 Rome, Italy; giuseppina.larosa@iss.it

**Keywords:** SARS-CoV-2, COVID-19, bivalve, shellfish, PCR, variant

## Abstract

Bivalve shellfish are readily contaminated by human pathogens present in waters impacted by municipal sewage, and the detection of SARS-CoV-2 in feces of infected patients and in wastewater has drawn attention to the possible presence of the virus in bivalves. The aim of this study was to collect data on SARS-CoV-2 prevalence in bivalve mollusks from harvesting areas of Campania region. A total of 179 samples were collected between September 2019 and April 2021 and were tested using droplet digital RT-PCR (dd RT-PCR) and real-time RT-PCR. Combining results obtained with different assays, SARS-CoV-2 presence was detected in 27/179 (15.1%) of samples. A median viral concentration of 1.1 × 10^2^ and 1.4 × 10^2^ g.c./g was obtained using either Orf1b nsp14 or RdRp/gene E, respectively. Positive results were unevenly distributed among harvesting areas and over time, positive samples being more frequent after January 2021. Partial sequencing of the spike region was achieved for five samples, one of which displaying mutations characteristic of the Alpha variant (lineage B.1.1.7). This study confirms that bivalve mollusks may bioaccumulate SARS-CoV-2 to detectable levels and that they may represent a valuable approach to track SARS-CoV-2 in water bodies and to monitor outbreak trends and viral diversity.

## 1. Introduction

The emergence and global spread of Severe-Acute Respiratory Syndrome Coronavirus 2 (SARS-CoV-2), responsible for the COVID-19 pandemic, poses an overwhelming challenge to health policies worldwide. SARS-CoV-2 is considered the third major coronavirus outbreak in the last 20 years, after Severe Acute Respiratory Syndrome (SARS) and Middle East Respiratory Syndrome (MERS) [1]. Coronaviruses (CoVs) are a group of enveloped viruses causing mainly respiratory and gastrointestinal tract infections. Most patients with coronavirus disease (COVID-19) present respiratory symptoms, while 18% exhibit gastrointestinal symptoms [2]. Some studies have reported the presence of viral RNA in the stools of COVID-19 patients in percentages ranging from 16.5% to 100% [3]. Furthermore, several studies have reported the detection of SARS-CoV-2 RNA in wastewater worldwide and the usefulness of the environmental surveillance to help public health officials to better understand the extent of SARS-CoV-2 spread in communities, to determine trends—increase or decrease—of the infection, and to provide an early warning for virus resurgence [4,5,6,7]. Using this approach, it becomes possible to monitor the epidemiology of virus infections even if they are not evident by clinical surveillance, especially because traditional epidemiological approaches may be limited by the asymptomatic nature of many viral infections and by under diagnosis of clinical cases [8].

Bivalve shellfish are filter-feeding organisms and are readily contaminated by human pathogens present in waters impacted by municipal sewage. The association of shellfish-transmitted infectious diseases with sewage pollution has been well documented since the late 19th and early 20th centuries [9]. Bivalve shellfish including oysters, clams, and cockles, are important vectors for viral enteric diseases. The risks related with consumption of shellfish are greater if these products are eaten raw or only partially cooked [10]. Relaying and depuration processes, which consist of placing shellfish in clean water for variable periods after harvesting from their production areas, are commonly used to reduce the amount of fecal bacteria in shellfish but do not efficiently eliminate viruses [11].

The detection of SARS-CoV-2 in the feces of symptomatic and asymptomatic infected patients, and consequently in wastewater, has drawn attention to the possible presence of the virus in the mollusks [12]. In fact, SARS-CoV-2 has been detected frequently in raw sewage and occasionally in treated sewage [13,14] and rivers [15,16], reinforcing the hypothesis that SARS-CoV-2 may reach the aquatic environment and mollusks may be contaminated in turn [17]. The low levels of virus contamination and the presence of PCR inhibitors in shellfish are the main obstacles for the application of RT-PCR for the detection of viruses in shellfish samples. Furthermore, sensitivity of the method is not always enough to detect the low viral concentration present in these types of environmental samples [18,19]. Dissections of the digestive tract and diverticula (hepatopancreas) appear to reduce the presence of inhibitors and to increase the sensitivity of such molecular methods [20], though the complete removal of PCR inhibitors cannot always be achieved.

Reverse transcription-polymerase chain reaction (RT-PCR) has emerged as the primary mode of diagnosis of acute infection with SARS-CoV-2, and a number of molecular targets have been identified, such as those coding for the nucleocapsid (N), the membrane protein (M), the envelope (E), and the spike (S) [21]. Open reading frames ORF1a and ORF1b and RNA-dependent RNA polymerase (RdRp), are some other genes that encode structural proteins that can be utilized for COVID-19 diagnosis [21]. It has been reported that in clinical practice the false-negative rate of real-time RT-PCR-based assays can be as high as 20% [22]. Several studies have shown that droplet digital PCR (ddPCR) has the advantages of absolute quantification and is more sensitive for virus detection than RT-PCR [23,24]. Digital PCR is based on the principles of limited dilution, end-point PCR, and Poisson statistics, with absolute quantification as its heart [25]. Therefore, quantification is less affected by poor amplification efficiency and inhibitors of amplification that may be present in samples.

The aim of this study was to collect data on the prevalence of SARS-CoV-2 in bivalve mollusks (*Mytilus galloprovincialis*) from harvesting areas of Campania region (Southern Italy) using dd RT-PCR for the detection of low levels of virus in mollusk tissues.

## 2. Materials and Methods

### 2.1. Sampling

A total of 179 samples were included in the monitoring: 150 samples were collected from bivalve mollusks farms sited in the Gulf of Naples (Campania region, Southern Italy), 3 were collected during operations of local health services to counter illegal harvesting, 2 samples—originating from Latina (Lazio, Italy) and Spain, respectively—were taken from fish shops in the city of Portici (Naples), and 24 samples were not traceable to specific production sites. Considering the samples originating from the coastal environment of Campania region, all the production areas included in the analysis of the present work were classified as class “B” according to Regulation (EU) 2019/675. Shellfish samples were collected from September 2019 to April 2021 and bivalves were kept at 4 °C during transport from the sampling point to the testing laboratory. Sample details included sampling date, time, sample type, vendor and address, sample temperature (ambient, refrigerated, frozen), sample origin/identification mark (if available); all data were recorded by sampling officers of local health authorities.

### 2.2. Sample Preparation

Viral recovery from mollusks was carried out according to the ISO 15216-2:2019, as bioaccumulation of SARS-CoV-2 in bivalve digestive tissue and suitability of the ISO method for Coronavirus recovery from oysters had been demonstrated in other studies [17]. For each sample, 2 g of digestive glands (hepatopancreas) homogenate was transferred to a clean tube and 10 µL of Mengovirus process control, strain vMC0 [26] were added to the samples to verify the efficiency of the extraction. Homogenates were then treated with 2 mL of a proteinase K solution (Qiagen, Hilden, Germany) to digest the tissue. The homogenates were incubated at 37 °C with shaking (approximately 320 rpm) for 60 min and, after that, at 60 °C for 15 min. The tubes were then centrifuged at 3000× *g* for 5 min and the supernatant was decanted into a new tube and measured.

### 2.3. Viral RNA Extraction

Total RNA was extracted from 500 µL of mollusks supernatant using the eGeneUP semi-automated system (bioMerieux, Marcy-l’Étoile, France) and the NucliSENS reagents (bioMerieux, Marcy-l’Étoile, France) following the manufacturer’s instructions, with the final elution in 100 µL of buffer. A negative extraction control sample (molecular biology water) was also processed and tested in parallel with each set of extracted samples.

### 2.4. Droplet Digital RT-PCR (dd RT-PCR) for SARS-CoV-2 Detection

SARS-CoV-2 detection was performed using three specific targets, the RdRp gene, the N gene [27], the Orf1b nsp14 region [28,29], and one target generic for beta-Coronavirus, the E gene [27]. The QX200 system (Bio-Rad, Hercules, CA, USA) was used to perform dd RT-PCR. The reaction (20 μL) included: 1X One-step RT-ddPCR Advanced Kit for Probes, 20U/μL Reverse transcriptase, 15 mM DTT, 5 μL of sample RNA, and nuclease-free water as required. Sequences and concentrations of primers and probes are reported in Table 1. The reaction mixtures were placed into the sample wells of DG8 cartridges (Bio-Rad) and a volume of 70 μL of droplet generation oil was loaded into the oil well. Droplets were formed in the droplet generator (Bio-Rad) and 40 μL of droplet-partitioned samples were transferred to a 96-well plate and sealed. The PCR amplification was carried out on a CFX96 Deep Well instrument (Bio-Rad) with the following thermal profile: 50 °C for 60 min, 95 °C for 10 min followed by 45 cycles of 95 °C for 15 s and 60 °C for 45 s, and a final stage at 98 °C for 10 min. After thermal cycling, the 96-well plate was read in the QX200 Droplet Reader, based on positive droplets and according to the Poisson distribution. QuantaSoft software was used to count the PCR-positive and PCR-negative droplets to provide absolute quantification of target DNA. The quantification of each target was expressed as the number of copies per 1 microliter of sample RNA. Positive controls consisted of a SARS-CoV-2 RNA containing the target genes (E, N, Orf1ab, and RdRp), certified by Bio-Rad Laboratories (Bio-Rad). The Limit of Detection (LOD) obtained allows to detect until 1 g.c. (genomic copies)/µL for each target.

Mengovirus process control was analyzed and recovery was calculated as described in ISO 15216-2:2019.

### 2.5. Real-Time RT-PCR Assay Using the Quanty COVID-19v2

In order to compare dd RT-PCR results to one of the real-time RT-PCR reference assays for SARS-CoV-2 detection, the Quanty COVID-19v2 (Clonit, Milano, Italy), an assay targeting two sites of the N region (N1 and N2), was considered. The real-time RT-PCR was carried out according to the manufacturer’s instructions in a 15 µL reaction mixture with 5 µL of sample RNA. Internal controls were included in each PCR reaction for inhibition control; one negative and two positive controls (for N1 and N2 target, respectively, supplemented by the manufacturer) were used in each run. The real-time RT-PCR was performed using the QuantStudio 12 Flex system (Thermo Fisher Scientific, Waltham, MA, USA). The assay interpretation criteria were as follows: SARS-CoV-2 RNA was detected if amplification of N1 and/or N2 were achieved with Ct < 40, negative in the absence of amplification for both targets, and invalid when a significant reaction inhibition was detected. The declared LOD for this analytical assay was 8.15 copies/µL for N1 and 5.45 copies/µL for N2.

### 2.6. Molecular Characterization of Positive Samples

Molecular characterization of SARS-CoV-2 in samples that were positive in either one of the ddPCR assays or in real-time PCR was performed using the nested RT-PCR assays described in La Rosa et al. (2021b) [30] with slight modifications. Briefly, 10 μL of sample RNA were reverse transcribed and amplified using the SuperScript III One-Step RT-PCR System (Invitrogen, Carlsbad, CA, USA). Amplification reactions (PCR IDs 972, 974, and 979) were performed with the corresponding primers (400 nM) in a 25 μL volume. The following amplification conditions were used: reverse transcription at 45 °C for 20 min, denaturation at 94 °C for 2 min, and 35 cycles of 94 °C for 15 s, 58 °C for 30 s, 68 °C for either 30 s (PCR IDs 972 and 974) or 1 min and 45 s (PCR ID 979), and a final extension at 68 °C for 5 min. Nested PCR assays (PCR IDs 973, 975, and 980) were performed in 25 μL volume using the Phusion Hot Start II DNA Polymerase with GC buffer (Thermo Fisher Scientific, Waltham, MA, USA), 2 μL of the first PCR product, the corresponding primers (400 nM) and the following amplification conditions: denaturation at 98°C for 30 s, 45 cycles of 98 °C for 10 s, 62 °C for 30 s, 72 °C for either 30 s (PCR IDs 973 and 975) or 1 min (PCR ID 980), and a final extension at 72 °C for 10 min. Standard precautions were taken to avoid laboratory contamination.

Amplification products were observed on ethidium bromide-stained gel electrophoresis (1.5% agarose gel), purified using the GRS PCR and Gel Band Purification Kit (GRISP, Porto, Portugal) and sequenced on both strands (Eurofins Genomics, Ebersberg, Germany). Sequences were submitted to GenBank under the accession numbers OK036467-OK036471. The GISAID CoVsurver tool (https://www.gisaid.org/epiflu-applications/covsurver-mutations-app/, accessed on 2 September 2021) was used for mutation analysis.

## 3. Results

Quality assurance criteria were met for all tested samples (average recovery: 6.0%, range 1.5–10.0%). The screening of bivalve mollusks collected between September 2019 and April 2021 from the coastal area of the province of Naples (Italy) revealed the presence of SARS-CoV-2 RNA in 19 of the 179 samples (10.6%) analyzed by dd RT-PCR method. In detail, 11/20 samples displayed amplification of the Orf1b nsp14 region (concentration range: 0.13–4.1 genome copies (g.c.)/µL of RNA), 10/20 samples the amplification of RdRp target (0.12–7.6 g.c./µL of RNA), and 4/20 samples the amplification of the E target (0.2 to 0.8 g.c./µL of RNA); no samples showed the amplification of the N gene. These results corresponded to an estimated median concentration of SARS-CoV-2 in bivalve mollusks digestive tissue of 1.1 × 10^2^ g.c./g, range 7.8 × 10^1^ to 2.6 × 10^3^ g.c./g (Orf1b nsp14 region), 1.4 × 10^2^ g.c./g, range 7.2 × 10^1^ to 4.9 × 10^3^ g.c./g (RdRp gene), 1.4 × 10^2^ g.c./g, range 1.3 × 10^2^ to 5.0 × 10^2^ g.c./g (E gene).

Overall, one sample displayed the presence of the virus with all the PCR targets (Orf1b nsp14, RdRp, E genes; Table 2), three samples were positive by the RdRp and E genes, one using the Orf1b nsp 14 and the RdRp gene, and 14 samples were positive only by one target (either Orf1b nsp14 region or the RdRp gene). The real-time RT-PCR assay (Quanty COVID-19, Clonit), targeting two sites of the N gene, gave positive results on 11/179 samples (Table 2), all with Ct values above 35. Of these, only three samples corresponded to dd RT-PCR-positive samples. Thus, combining results obtained by dd RT-PCR and real-time RT-PCR analysis, the presence of SARS-CoV-2 was detected in 27/179 (15.1%) samples.

**Figure 1:**
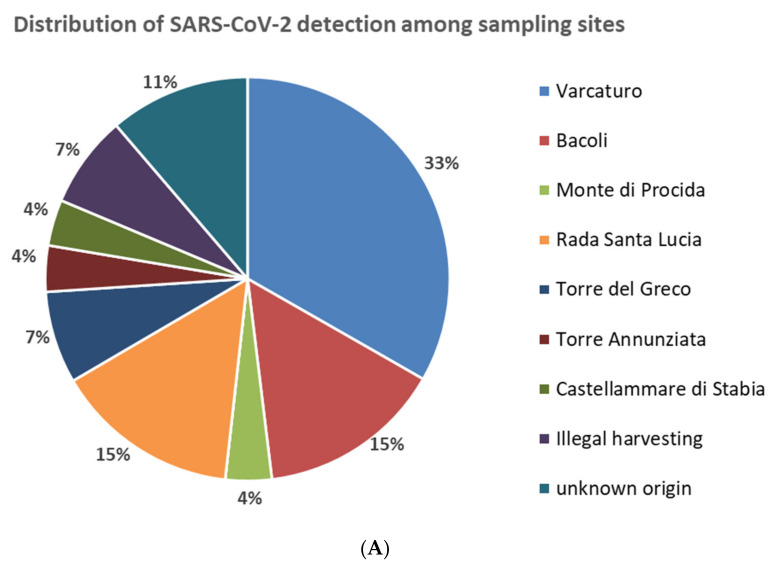

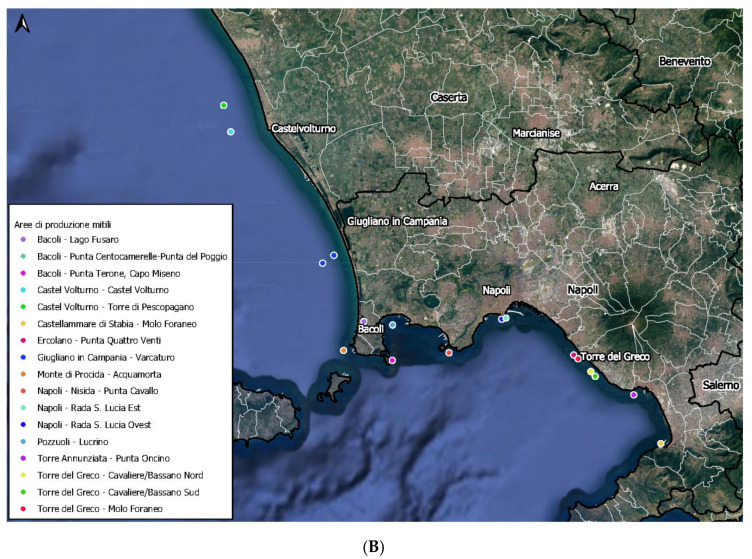
(**A**) Percentage of SARS-CoV-2 positive samples according to sampling sites. (**B**) Distribution of sampling sites in the Gulf of Naples (Campania region, Italy).

Positive samples were collected from seven bivalve mollusks farms in the area of Gulf of Naples; other positive results were associated to bivalve shellfish taken from non-authorized areas illegally used for shellfish harvesting or were of unknown origin (Figure 1 and Table 3). The production areas showing the highest number of positive samples were located in the north and center part of the coastline of region Campania. In detail, 9/27 (33%) of positive samples came from the site ‘Varcaturo’, 4/27 (15%) came from the site ‘Rada Santa Lucia’ and 4/27 (15%) from the site ‘Bacoli’ (Figure 1). The sample with the highest viral concentration by dd RT-PCR of the RdRp gene (7.6 g.c./µL) was collected in February 2021 in a farm located in the site ‘Monte di Procida’ (Figure 1).

**Figure 2:**
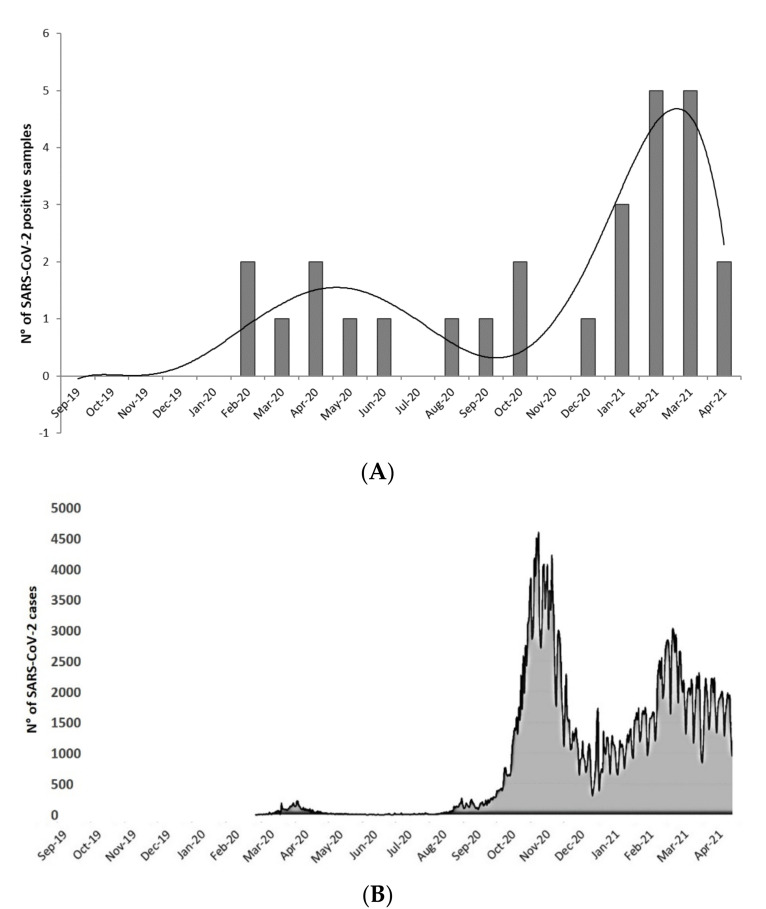
(**A**) Trend of SARS-CoV-2 detection in bivalve mollusks samples included in the monitoring (September 2019 to April 2021). (**B**) SARS-CoV-2 infection trend (daily new cases in Campania region residents from September 2019 to April 2021. Modified from Ministry of Health; https://public.flourish.studio/story/722265/) (accessed on 23 July 2021).

SARS-CoV-2 RNA was detected at a higher frequency in the samples collected in the period between January and March 2021 (Figure 2A). In detail, detection was achieved in 3 of 8 samples (38%) collected in January 2021, 5 of 11 (45%) in February, and 5 of 16 (31%) in March. These results were consistent with regional epidemiological data (data source: Health Minister; https://public.flourish.studio/story/722265/ (accessed on 7 January 2022)), which displayed a high increase of daily new cases in October and November 2020, and then a less pronounced increasing trend from January to March 2021 (Figure 2B).

Regarding confirmation of dd RT-PCR and real-time RT-PCR positive samples and their molecular characterization by nested RT-PCR, amplification of SARS-CoV-2 partial spike region was achieved in 5 of the 27 tested samples (Table 2): samples IDs 118, 119, 177, and 178 were positive by PCR 973, sample ID 101 by PCR ID 975, while no amplification was obtained with PCR 980. All amplified samples were collected between January and April 2021. Sequenced fragments of samples 118, 119, 177, and 178 (aa 58–150 of the spike gene) displayed 100% identity to the corresponding region of prototype sequence NC_045512 (Wuhan strain). On the other hand, sample ID 101 (aa 480–573 of the spike gene), collected in March 2021, displayed the mutations N501Y and A570D characteristic of SARS-CoV-2 Alpha variant (B.1.1.7).

## 4. Discussion

The aim of the present study was the investigation of SARS-CoV-2 presence in bivalve shellfish of Campania region, in order to ascertain virus prevalence and concentration and investigate the possible use of shellfish for monitoring the spread of SARS-CoV-2. The coastal environment is subjected to contamination by a large variety of human viruses from sewage, which can be bio-accumulated by filter-feeding shellfish species. Possible contamination by SARS-CoV-2 of coastal waters and other environmental compartments, such as estuaries and marine habitats, has been hypothesized by some authors, as well as the possibility to use shellfish as sentinels of their environment [12,17]. Although monitoring of SARS-CoV-2 in sewage discharges should be considered as the first choice for biocontrol, bivalves could be used as a surveillance tool, especially in case of non-point sources, direct wastewater discharges in small estuaries, and difficulties in sampling [31]. In addition, the continuous filter-feeding activity of bivalve mollusks, modeling their ability to bioaccumulate chemical and microbial contaminants, may help overcome the limitations of wastewater analysis deriving from low concentration of the viral target and limited analytical sensitivity. Some species such as mussels (*Mytilus* spp.) are already widely used as bioindicators for coastal water pollution monitoring [32]. For these reasons, bivalves have good features to be considered sentinels for the detection of SARS-CoV-2 in the coastal environment.

It should be noted that the existence of RNA molecules binding with biofilm matrices provides a prolonged persistence of RNA in the environment (eRNA) and contributes to the conservation of eRNA in the marine environment much longer than expected [33,34], supporting the use of environmental analysis to trace viral spread and genetic variability. However, in a risk assessment perspective, it should be considered that while SARS-CoV-2 RNA may be stable in river and sea waters, the presence of RNA alone does not correlate infectious virus [14,35,36]. A previous study on SARS-CoV-2 in bivalve shellfish from Polo et al. (2021) [12] using PCR viability assays, showed that real-time detection of SARS-CoV-2 RNA in clams and sediments did not correspond to intact capsids and, therefore, to infectious viral particles, and concluded that the risk to public health associated to SARS-CoV-2 in bivalve shellfish is extremely low, so that shellfish should not be inappropriately perceived as a risk or a vector of SARS-CoV-2. Moreover, although the presence of SARS-CoV-2 RNA in wastewater influents has been confirmed, viral infectivity of positive samples in cell cultures has not been proven so far [37]. Respiratory droplets and aerosols may contain high titers of viral particles [38,39,40] and SARS-CoV-2 infectivity is retained for over 3 h in experimentally produced aerosols [41]. On the contrary, occurrence of infectious virus in feces and urine has been questioned: detection of SARS-CoV-2 in human feces (reviewed in Foladori et al. 2020 [42]) is indeed a common feature in infected subjects, but isolation of infectious virus from this clinical specimen has been achieved in a limited number of cases [43,44,45,46], while other studies failed to do so [47,48,49]. Therefore, it may be hypothesized that feces and urine probably contain either low levels or no infectious SARS-CoV-2 particles and that, based on low predicted abundances and limited environmental survival, the likelihood of SARS-CoV-2 transmission though sewage-contaminated water or bivalve shellfish is extremely low or negligible [50].

In this study we chose to apply the current recommended method ISO15216-1:2019 for the detection of Norovirus and hepatitis A in mollusks, adapting it to SARS-CoV-2, given the existing evidence for a higher concentration of this virus in the digestive tissue of bivalve shellfish [17]. In the absence of a reference method for SARS-CoV-2 testing in foods, different analytical approaches (dd RT-PCR and real-time RT-PCR) and protocols based on different genetic targets (Orf1b nsp14 region, RdRp, E gene, and N gene) were included in the study, to provide a better estimate of SARS-CoV-2 prevalence. Further to this, to unambiguously confirm detection of SARS-CoV-2 and perform molecular characterization, all positive results obtained by dd RT-PCR or real-time RT-PCR were subjected to conventional nested RT-PCR followed by sequencing.

Overall, presence of SARS-CoV-2 was shown in 27/179 (15.1%) of samples, with a median viral concentration in positive samples of ~10^2^ g.c./g, regardless of the applied assay. These results confirm, though with a significantly lower prevalence, the study by Polo et al. (2021) [12] on SARS-CoV-2 occurrence in clams and sediments collected from estuarine areas in Galicia (Spain) between May and July 2020, in which SARS-CoV-2 RNA was detected in 9/12 clam and 3/12 sediment samples. On the contrary, a similar survey conducted on oysters taken between April and August 2020 from several areas of the French coasts reported no detection of SARS-CoV-2 in over 180 samples [17].

Results from these studies, however, should be considered taking into account the concentration of the viral target in the shellfish matrix. Viral concentration in bivalve shellfish may be significantly affected by the viral load in the growing waters, which is a function of both viral input through wastewater discharge and virus dispersion in seawaters due to dilution effect and water circulation. The two studies undertaken in France and Spain were conducted on samples collected in spring and summer 2020, in correspondence to the so-called first wave of the COVID-19 pandemic. Our study encompassed a longer observation period (September 2019 to April 2021) and highlights that, even though SARS-CoV-2 was detectable since February 2020 (i.e., at the beginning of the COVID-19 pandemic in Italy) and remained stable during the entirety of 2020, the number of positive samples increased since January 2021, following the so-called second wave of the epidemic. This result probably reflects an increase of viral discharge in wastewaters, and consequently in seawaters, associated to the increase of infection cases in the population of the region Campania, as indicated by the trends reported by the national surveillance system for COVID-19.

Sewage discharge, however, may impact bivalve shellfish harvesting areas to a different level, based on viral particle dispersion in seawater (distance from the contamination source, dilution effects, water streams, tide effects, etc.). In our study, production areas along the whole coastline of region Campania (90 km) were included and a significant variability in SARS-CoV-2 detection was seen among relatively close areas of the northern part of the coastline, where virus was detected in 41% of samples from the site ‘Varcaturo’ (the closest site to the major wastewater treatment plants of the region), in 15% of those taken in ‘Bacoli’, and never detected in the sites ‘Pozzuoli’ and ‘Nisida’. SARS-CoV-2 was also detected in a significant proportion of samples collected in the site ‘Rada Santa Lucia’, the closest to the urban center of Naples. Indeed, the sampling sites where SARS-CoV-2 was detected in bivalve mollusks represent the meeting points of multiple sewer systems. The positivity revealed on “Rada Santa Lucia”, for example, may be attributed to the contribute of a wide area with a high population density. In this site the treated wastewater coming from two sewer systems contributes together to generate the exposure on mussels.

On the other hand, virus was detected in a minority of samples collected in the three sites in the southern part of the coastline. These results highlight that high variability may be expected for SARS-CoV-2 detection depending on the characteristics of the sampling sites and may shed light on the differences in SARS-CoV-2 prevalence and concentration in our study, performed mostly on mussels (*Mytilus galloprovincialis*) grown in areas located approximately within 3 km from the coast, compared to the work by Polo et al. (2021) [12], which tested clams (*Ruditapes philippinarum*) grown in banks located in small estuaries influenced by tides and relatively close to wastewater treatment plants or sewage pump stations.

Further to the variables associated to SARS-CoV-2 concentration in growing waters, analytical issues may affect SARS-CoV-2 detection in complex food matrices such as bivalve shellfish. In our study, viral concentration was, with few exceptions, below 1 g.c./µL of tested RNA, hence in a concentration range close to the detection limit of molecular tests, a factor that—due to statistical probability—may affect target detection. This effect, together with the intrinsic variability of amplification efficiency of different PCR assays, partially explain the inconsistency of the results obtained with the different protocols and the fact that only a minority of samples (5 of 19 considering dd RT-PCR results) were positive by two or more assays.

Interestingly, despite the low target concentration, amplification by RT-nested-PCR and molecular characterization by partial sequencing of the spike gene was achieved in five samples. In all such cases, amplification was obtained using PCR assays for short fragments (PCR IDs 973 and 975, ~320 bps) but not with the assay designed to amplify ~1600 bps of the spike gene (PCR ID 980). This result is not surprising, as amplification of long fragments in complex matrices as foods or environmental samples is often challenging, due to low target concentration and fragmentation of nucleic acids. Significantly, the obtained amplifications showed 100% sequence identity to SARS-CoV-2 prototype strain (Wuhan sequence) in four samples collected between January and April 2021, but also displayed two mutations (N501Y and A570D) typical of the so-called ‘UK variant’, later renamed ‘Alpha variant’ according to WHO nomenclature (https://www.who.int/en/activities/tracking-SARS-CoV-2-variants/, accessed on 5 September 2021), in one sample collected in March 2021, i.e., less than three months after the first isolation, on December 2020, of this Variant of Concern (VOC) in Italy (https://press.regione.puglia.it/-/covid-l-istituto-zooprofilattico-ha-isolato-il-virus-della-cosiddetta-variante-inglese, accessed on 5 September 2021).

## 5. Conclusions

To our knowledge, this is the first study providing molecular characterization of SARS-CoV-2 in bivalve shellfish and reporting the detection of mutations characteristic of a VOC.

In conclusion, this study highlights that bivalve mollusks may bioaccumulate SARS-CoV-2 to levels detectable by droplet digital RT-PCR or by real-time RT-PCR and that detection rate varies over time in connection to viral shedding in the growing waters. Further to this, molecular characterization and variant identification of SARS-CoV-2 by sequencing may be achieved in positive shellfish samples. Therefore, bivalve mollusks may represent an interesting approach to track SARS-CoV-2 spread in water bodies and a valuable alternative to monitor outbreak trends and viral diversity.

## Figures and Tables

**Table 1:** Sequence, concentrations, and reference of primers and probe used in this study.

Primer Name	Sequence	Concentrations	Reference
RdRp_SARSr-F	GTGARATGGTCATGTGTGGCGG	600 nM	Corman et al., 2020
RdRp_SARSr-R	CARATGTTAAASACACTATTAGCATA	800 nM	
RdRP_SARSr-P1	FAM-CCAGGTGGWACRTCATCMGGTGATGC-BBQ	100 nM	
RdRP_SARSr-P2	FAM-CAGGTGGAACCTCATCAGGAGATGC-BBQ	100 nM	
N_Sarbeco_F	CACATTGGCACCCGCAATC	600 nM	Corman et al., 2020
N_Sarbeco_R	GAGGAACGAGAAGAGGCTTG	800 nM	
N_Sarbeco_P	FAM-ACTTCCTCAAGGAACAACATTGCCA-BBQ	200 nM	
2297-CoV-2-F	ACATGGCTTTGAGTTGACATCT	500 nM	La Rosa et al., 2021
2298-CoV-2-R	AGCAGTGGAAAAGCATGTGG	900 nM	
2299-CoV-2-P	FAM-CATAGACAACAGGTGCGCTC-MGBEQ	250 nM	
E_Sarbeco_F	ACAGGTACGTTAATAGTTAATAGCGT	400 nM	Corman et al., 2020
E_Sarbeco_R	ATATTGCAGCAGTACGCACACA	400 nM	
E_Sarbeco_P1	FAM-ACACTAGCCATCCTTACTGCGCTTCG-BBQ	200 nM	

FAM: 6-Carboxyfluorescein; BBQ: blackberry quencher; MGB: minor groove binder; EQ: Eclipse quencher.

**Table 2:** Summary of ddPCR (A) and real-time RT-PCR (B) study results. (A) Summary of dd RT-PCR results. (B) Summary of real-time RT-PCR results.

**(A)**					
**dd RT-PCR Gene Target**					**N° of Sequenced Samples**
**N° of Samples**	**Orf1b nsp 14 Region**	**RdRp Gene**	**E Gene**	**N Gene**	
1	+	+	+	-	1
1	+	+	-	-	0
3	-	+	+	-	0
9	+	-	-	-	1
5	-	+	-	-	2
160	-	-	-	-	1
179	11	10	4	0	5
(**B**)					
**Real-Time RT-PCR Gene Target**					**N° of Sequenced Samples**
**N° of Samples**	**N1**		**N2**		
1	+		+		0
5	+		-		3
5	-		+		0
168	-		-		2
179	6		6		5

**Table 3:** Detection of SARS-CoV-2 in bivalve mollusk samples according to sampling site.

Sampling Site	Position in the Coastline	SARS-CoV-2 Detection		
		N° of Positive Samples	% of Positive in the Site	% of Positive in the Total
Varcaturo	North	9/22	41	5.0
Bacoli	North	4/27	15	2.2
Monte di Procida	North	1/14	7	0.6
Pozzuoli	North	0/8	0	0
Nisida	North	0/10	0	0
*Subtotal North*		14/81	17	7.8
Rada Santa Lucia	Center	4/16	25	2.2
*Subtotal Center*		4/16	25	2.2
Torre del Greco	South	2/32	6	1.1
Torre Annunziata	South	1/7	14	0.6
Castellammare di Stabia	South	1/14	7	0.6
*Subtotal South*		4/53	8	2.2
Other origins		0/2	0	0
Illegal harvesting	-	2/3	67	1.1
Unknown origin		3/24	13	1.7
*Total*		27/179	-	15.1%

## Data Availability

The data presented in this study are available on request from the corresponding author.
